# Mitochondrial ribosomal protein S18-2 is highly expressed in endometrial cancers along with free E2F1

**DOI:** 10.18632/oncotarget.7905

**Published:** 2016-03-03

**Authors:** Miriam Mints, Muhammad Mushtaq, Natalia Iurchenko, Larysa Kovalevska, Maria C Stip, Daria Budnikova, Sonia Andersson, Ludmila Polischuk, Lubov Buchynska, Elena Kashuba

**Affiliations:** ^1^ Department of Women's and Children's Health (KBH), Karolinska Institutet, Stockholm, 17176, Sweden; ^2^ Department of Microbiology, Tumor and Cell Biology (MTC), Karolinska Institutet, Stockholm, 17177, Sweden; ^3^ R.E. Kavetsky Institute of Experimental Pathology, Oncology and Radiobiology, Kyiv, 03022, Ukraine

**Keywords:** endometrial cancer, mitochondrial ribosomal protein MRPS18-2, cell transformation, E2F1, prognostic markers

## Abstract

Endometrial cancer (EC) is one of the most frequent causes of cancer death among women in developed countries. Histopathological diagnosis and imaging techniques for EC are limited, thus new prognostic markers are needed to offer patients the best treatment and follow-up.

In the present paper we showed that the level of mitochondrial ribosomal protein MRPS18-2 (S18-2) increased in EC compared with the normal endometrium and hyperplasia, based on a study of 42 patient biopsies. Importantly, high expression of free E2F1 in EC correlates well with high S18-2 expression. The EC cell line HEC-1-A, which overexpresses S18-2 constitutively, showed an increased proliferation capacity *in vitro* and *in vivo* (in SCID mice). Moreover, pan-keratin, beta-catenin and E-cadherin signals are diminished in these cells, compared to the parental HEC-1-A line, in contrast to vimentin signal that is increased. This may be associated with epithelial-mesenchymal cell transition (EMT).

We conclude that high expression of S18-2 and free E2F1, and low pan-keratin, beta-catenin, and E-cadherin signals might be a good set of prognostic markers for EC.

## INTRODUCTION

Endometrial cancer (EC) accounts for about 6% of all cancer in females worldwide and is one of the most frequent causes of cancer death among women in developed countries. The number of new cases of EC has gradually risen since 1960, and EC now accounts for 40% of gynecological cancers worldwide. Overweight, hypertension, physical inactivity, diabetes, and family history are among the most important risk factors for EC [1].

EC originates in the endometrial lining of the uterus. Up to 10% of EC develops into an aggressive form of the disease, with 5-year survival rates between 5% and 10% [2]. All women with EC who are considered operable undergo hysterectomy and bilateral salpingophorectomy. In cases with aggressive tumor histology, such as low differentiation or aneuploidy, a more extensive procedure is recommended, such as pelvic and paraortal lymph node evacuation and omentectomy [3]. Current imaging techniques like CT, MRI and PET have low sensitivity and specificity to locate endometrial tumors and their depth of invasion into the myometrium and cervix, or lymph node metastases. Current recommendations for surgical treatment are based on the histology of the endometrial biopsy. Histopathology does not reflect the molecular properties of tumors [4]. Thus, the current limitations of histopathological diagnosis and imaging techniques of EC require the development of biomarkers that reflect the molecular functional profile of endometrial tumors. Identification of new prognostic markers is important as it will allow us to offer the best treatment and follow-up to EC patients [5].

The mitochondrial ribosomal protein S18-2 (MRPS18-2, **NP_054765**; S18-2 later in the text) might be considered as one of the possible markers. Overexpression of this protein in primary rat fibroblasts resulted in immortalization and eventual transformation of cells, as we found recently. Immortalized cells showed a phenotype resembling that of stem cells [6, 7]. Moreover, chromosomal instability was induced in transformed cells, which gave rise to tumors in SCID mice [8]. We have also shown previously that S18-2 protein binds RB (RB1, **NP_000312**), consequently freeing E2F1 (**NP_005216**) [9, 10]. Thus, it seems clear that overexpression of S18-2 provides a permanent stimulus for cell division, suggesting that it is involved in carcinogenesis.

To explore this, in the present paper we studied expression of S18-2 and free E2F1 proteins in EC, hyperplasia (HP), and the normal endometrium (NE). We also studied the influence of a high S18-2 expression on levels of anti-keratin, beta-catenin, E-cadherin, and N-cadherin in the EC-derived HEC1-A cell line and sub-line that expressed S18-2 constitutively at high levels.

## RESULTS

### Characteristics of patient groups

ECs were graded based on morphological features, according to the criteria of the 2009 International Federation of Gynecology and Obstetrics (FIGO) [11] and placed in six tissue categories: i) highly differentiated adenocarcinoma (HDA, FIGO grade 1); ii) moderately differentiated adenocarcinoma (MDA, FIGO grade 2); iii) minimally differentiated adenocarcinoma (LDA, FIGO grade 3); iv) serous cancers (SC); v) HP; and vi) NE.

Some tissue samples contained different histological areas, for example, adenocarcinoma and HP, serous tumor and HP, etc. Age and body mass index (BMI) distribution were studied for all women. Age (Figure S1A) and BMI (Figure S1B) did not differ significantly across the six groups of patients. Influence of the number of births and tumor recurrence were not considered in the present paper.

Expression of progesterone receptor (PR) and estrogen receptor (ER, ESR1, **NP_000116**) was also analyzed in tissues. As was expected, PR expression tended to decrease with tumor progression (Figure S1C), while ER expression was high in all samples of tumor tissue (Figure S1D).

### Expression of S18-2 protein is increased in tumors, compared with normal tissues and hyperplasia

**Figure 1:**
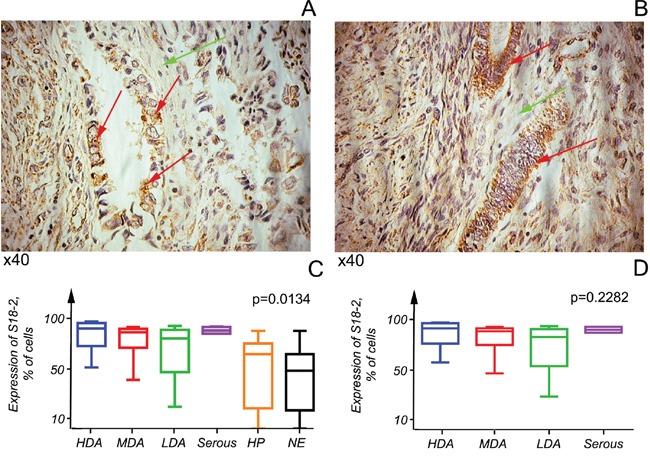
S18-2 protein expression in cancerous and normal cells S18-2 signal (in brown) is elevated in cancerous cells (red arrows) in comparison with stromal cells (green arrows). Highly differentiated tumors are shown **A, B.** Kruskal–Wallis tests were applied to six tissue categories, comprising three endometrioid adenocarcinomas at various differentiation stages and serous carcinoma, hyperplasia, and normal endometrium. In all tumors S18-2 is expressed at a significantly higher level than in hyperplasia (HP) or normal endometrium (NE) (p<0.05) **C.** No significant differences (p>0.05) in expression of two types of tumors were observed. Notice the tendency of the S18-2 signal to decrease in advanced endometrioid adenocarcinomas **D.**

S18-2 signal was almost absent in NE samples and very weak in HP. In contrast, EC demonstrated a strong S18-2 signal in cytoplasm (Figure 1). It is worth noting that a proportion of cancerous cells in LDA and SC showed a nuclear S18-2 signal. However, this proportion did not exceed 5% of all tumor cells. An example of S18-2 staining in EC samples are shown on Figure 1A and 1B.

We observed a significant difference (p<0.05) in S18-2 expression in all EC samples and in HP and NE samples combined (Figure 1C). Interestingly, S18-2 expression was high in all tumor tissue categories, but LDA tended to show decreased S18-2 expression (Figure 1D). This expression pattern may be connected to the stability of the S18-2 protein, which is largely unknown.

### Expression of the free E2F1 is increasing with the tumor grade

**Figure 2:**
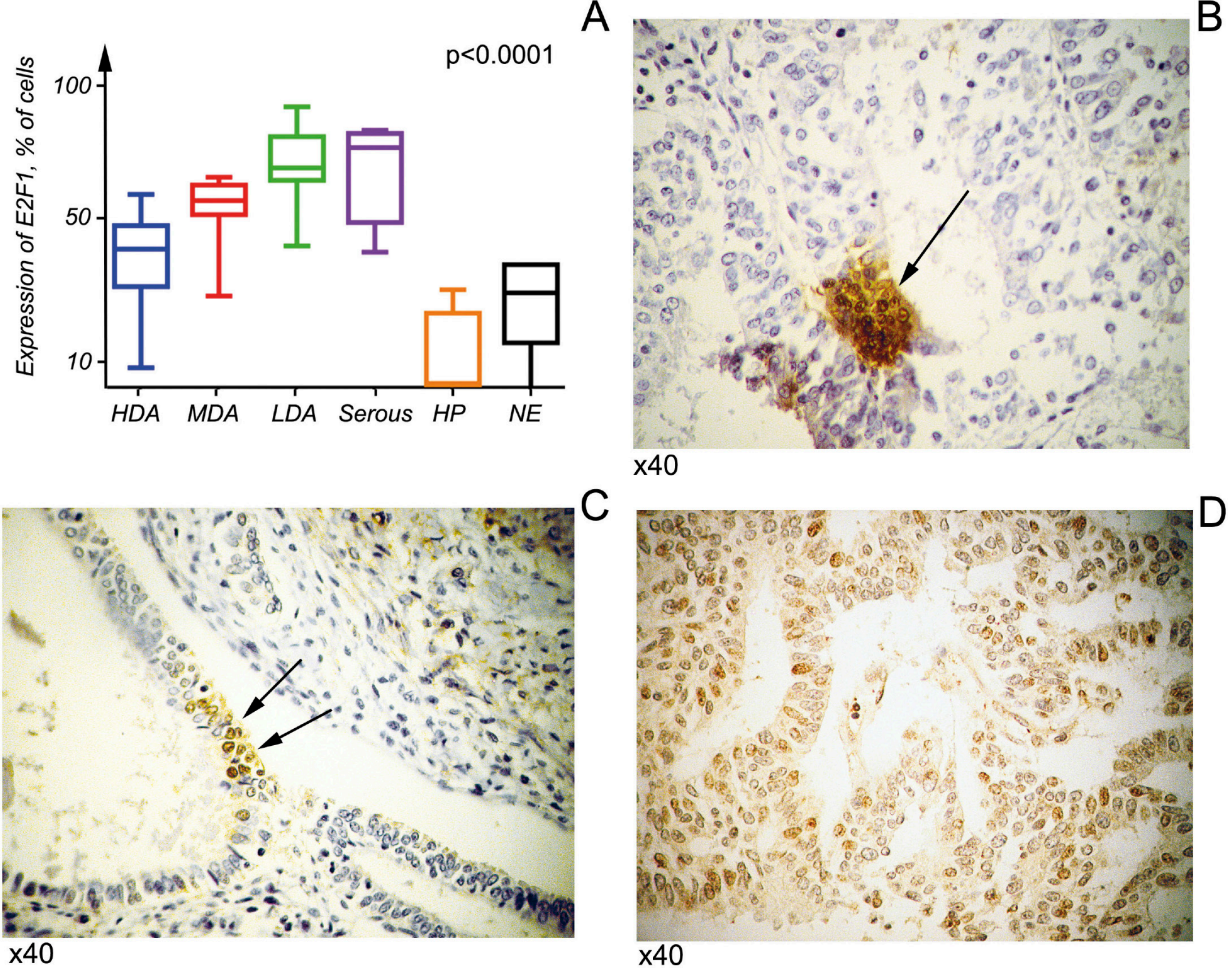
Free E2F1 expression in cancerous and normal cells Kruskal–Wallis tests were applied to six groups, comprising three of endometrioid adenocarcinomas at various differentiation stages and serous carcinoma, hyperplasia, and normal endometrium. In all tumors E2F1 showed a significantly higher signal than in hyperplasia or normal endometrium (p<0.05) **A.** Notice that the nuclear E2F1 signal was lower in more differentiated tumors, HDA **B, C.** compared with more advanced tumor, MDA **D.**

Due to the fact that S18-2 protein levels were elevated in tumor samples, it was of great importance to assess expression of the free E2F1 protein. To do so, the antibody raised against the C-terminus of E2F1 (residues 409-426) was used. It was shown that RB protein binds the C-terminal portion of E2F1 and this binding abolishes the transactivating ability of the latter [12, 13]. The signal of the free E2F1 was elevated in less differentiated tumors, and it was significantly higher than in NE and HP (Figure 2). The high free E2F1 expression in tumors correlates well with highly expressed S18-2.

### Overexpression of S18-2 in HEC-1-A EC cells induces epithelial-mesenchymal cell transition (EMT)

**Figure 3:**
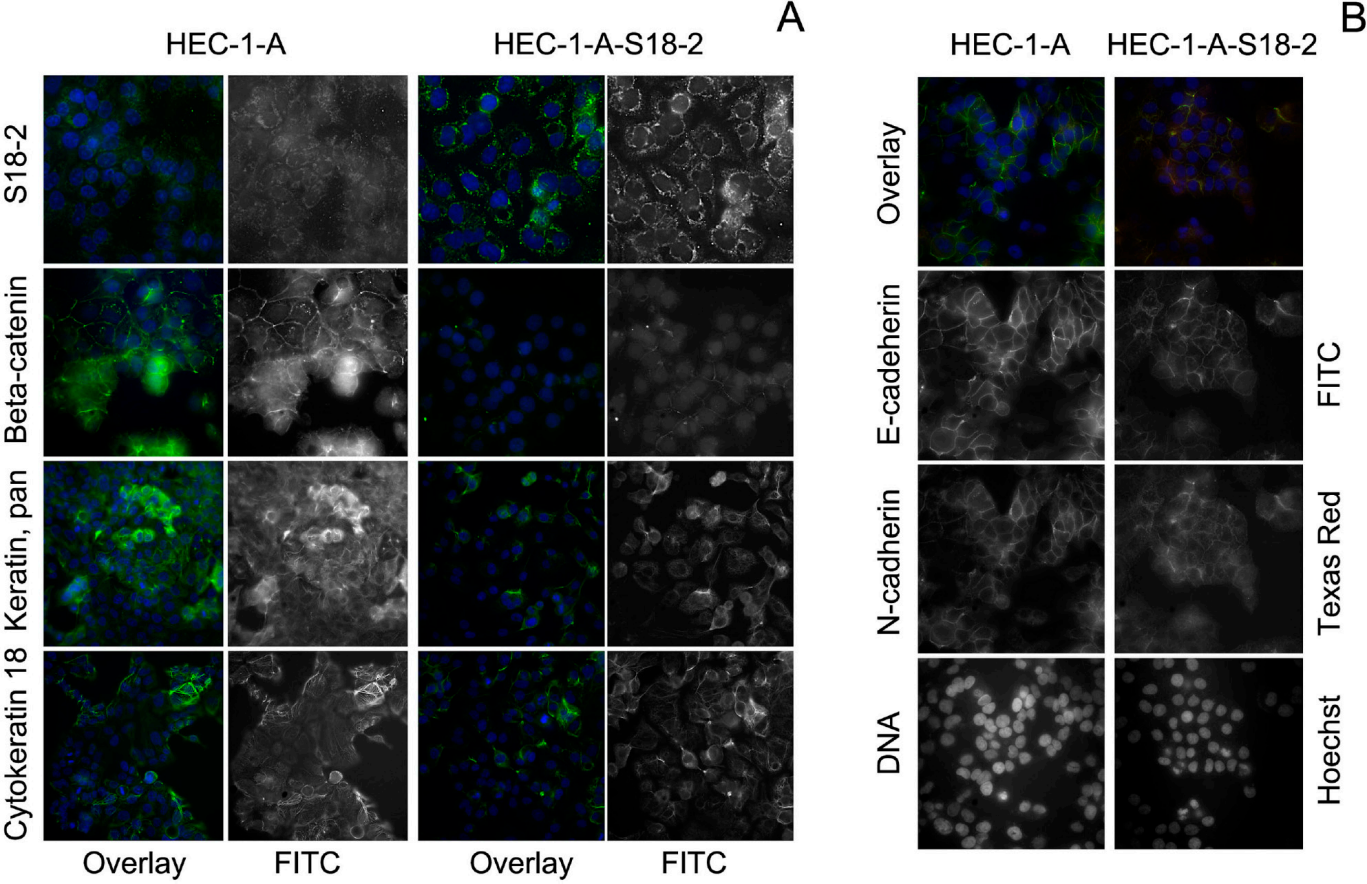
Expression pattern of S18-2, beta-catenin, pan-keratin, cytokeratin 18, E- and N-cadherins in HEC-1-A-S18-2 cells compared with HEC-1-A Notice the strong signal of ectopic S18-2 in HEC-1-A-S18-2 cells, compared to endogenous signal in original cell line (green on the merged images, white when single). Notice the dramatic reduction of beta-catenin, pan-keratin and cytokeratin 18 expression in HEC-1-A-S18-2 cells. DNA is shown in blue **A.** Notice the significant reduction of E-cadherin expression in HEC-1-A-S18-2 cells, compared to original cell line (green on the merged images, white when single). DNA is shown in blue on the merged images and white when alone **B.** Images were taken with objective x63.

**Figure 4:**
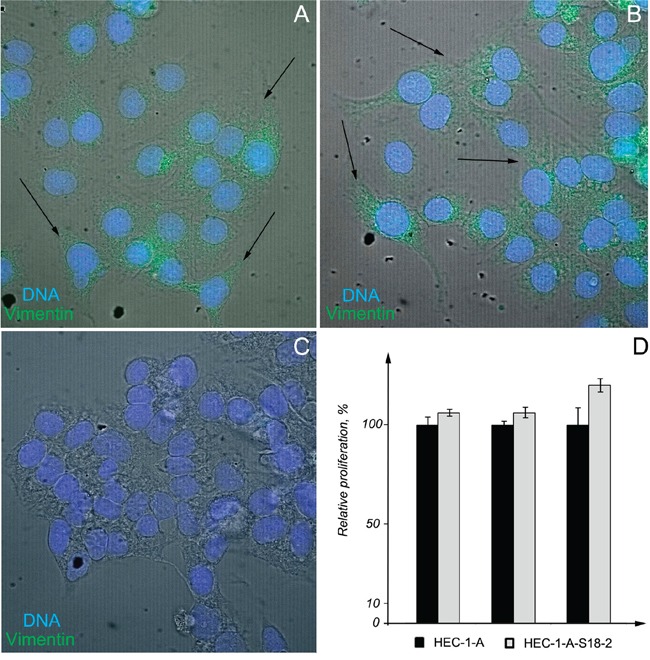
Pattern of vimentin expression and proliferation of HEC-1-A-S18-2 cells compared with HEC-1-A Signals of vimentin (green) and DNA (blue) were overlaid with the phase contrast (grey). Images were taken with objective x63. Notice the fibroblast-like morphology of HEC-1-A-S18-2 cells (indicated by arrows) **A, B.** and elevated vimentin signal in them **(A, B)** compared with parental HEC-1-A cells **C.** Notice the increased proliferation of HEC-1-A-S18-2 cells compared to parental HEC-1-A cells **D.** It is shown by percentage of increase in intensity of fluorescent signal that is proportional to cell number. The experimental value for HEC-1-A cells was taken as 100%. Experiments were performed for 500, 1000 and 3000 cells for each cell line in triplicates. Here the mean values and standard deviations are shown.

As was described above, tumors tissues showed a higher S18-2 signal. To find out the molecular changes in EC cells, HEC-1-A line produced from EC and its sub-line that overexpressed ectopical S18-2 (HEC-1-A-S18-2) were compared. Levels of S18-2 protein were assessed by immunostaining with anti-S18-2 antibody (Figure 3A, the top panel). To compare cell properties, expression pattern of pan-keratin, cytokeratin 18, E-cadherin, N-cadherin, beta-catenin, and vimentin was studied by immunofluorescent analysis. The decrease in the signals of pan-keratin, cytokeratin 18, beta-catenin, and E-cadherin and increase of vimentin signal was observed in the HEC-1-A-S18-2 cells that expressed S18-2 constitutively at the high level, as compared with the parental cells (Figures 3 and 4). Levels of N-cadherin were either unchanged or decreased in HEC-1-A-S18-2 cells (Figure 3B). Contrary, vimentin was stained only in HEC-1-A-S18-2 cells (Figure 4A and 4B). Noteworthy, the latter showed more “fibroblast-like” morphology (Figure 4A and 4B). This suggests that cells undergo EMT upon overexpression of S18-2, which is usually associated with an enhanced invasion ability of tumor cells. The proliferation capacity was assessed for both, parental HEC-1-A cells and the sub-line that expresses S18-2 constitutively at the high levels. Increase in relative proliferation was detected in HEC-1-A-S18-2 cells (Figure 4D).

### Expression of S18-2 in HEC-1-A cells increases the rate of tumor growth in SCID mice

**Figure 5:**
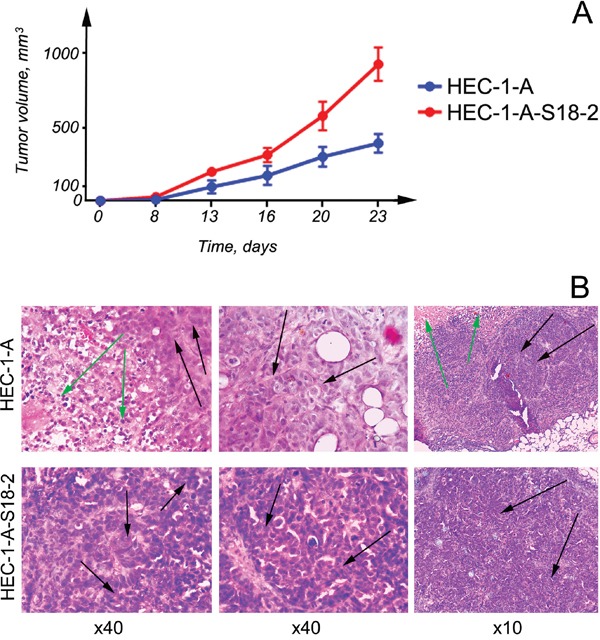
Proliferation of cells that were inoculated into SCID mice subcutaneously Notice that HEC-1-A-S18-2 cells produced larger tumors than parental HEC-1-A cells **A.** When HEC-1-A cells were introduced into SCID mice (the top panel), necrotic area (indicated by green arrows) and also well developed gland-like structures (black arrows) was developed. When HEC-1-A-S18-2 cells were inoculated, they formed more nicely formed foci (indicated by arrows) **B.**

Based on the immunochemistry experiments and in order to characterize the tumorigenicity of the HEC-1-A and the HEC-1-A-S18-2 cell line, cells were injected subcutaneously into SCID mice. Tumors were found in 100% (8 spots out of 8 injections) of the experimental animals and were detected 1-2 weeks after inoculation. All experimental animals were sacrificed at the same time on day 23, when the largest tumor reached 1 cm^3^, as stated in a protocol and approved by the Ethical committee. After analysis of growth curves, we concluded that the HEC-1-A-S18-2 cell line proliferated faster than the primary HEC-1-A cells (Figure 5A).

HEC-1-A cells, as expected, gave rise to well differentiated endometrioid carcinomas in experimental animals (Figure 5B, the top panel). Gland-like structures (indicated by black arrows) and necrotic areas (green arrows) appeared in tumors that developed in just 3 weeks. When HEC-1-A-S18-2 cells proliferated in SCID mice, the resultant tumors showed similar features, i.e., areas of necrosis and gland-like structures (Figure 5B, the bottom panel). However, these tumors were different in that they had a large number of blood vessels and more isolated gland-like structures, forming foci (indicated by arrows). To the naked eye, such tumors looked more differentiated than those obtained by inoculation of parental HEC-1-A cells.

## DISCUSSION

EC is divided into two types. Type 1 EC is morphologically classified as endometrioid carcinoma. It accounts for 80% of all cases and affects both pre- and post-menopausal women. Type 2 EC is morphologically classified as serous or clear cell carcinoma and affects older, postmenopausal women. Type 2 EC develops directly from the endometrium without hyperplasia. These tumors are often poorly differentiated and have a worse prognosis. Type 1 EC is characterized by a variety of genetic alterations, the most frequent of which are mutations in the tumor suppressor gene *PTEN* (**NM_000314**). *PTEN* mutations have been observed in up to 83% of endometrioid carcinomas and 55% of precancerous endometrial lesions [14–16].

The high TP53 (**NP_000537**) expression is a good prognostic marker for type 1 EC, it is higher in grade 3 than grade 1 tumors or NE tissue [17, 18]. The TP53 is mutated in only 10 to 15% of EC [19]. Earlier we showed that high TP53 expression is inversely correlated with MDM2 (**NP_001138809**) expression, which suggests that TP53 is not functional in endometrioid adenocarcinomas [20]. Although the mechanism of this stabilization has not yet been revealed, it may be linked to the high level of ER in endometrioid adenocarcinoma.

Contrary to type 1 EC, *TP53* is mutated in about 90% of type 2 EC, such as serous carcinoma. Other frequent genetic alterations in type 2 EC are inactivation of p16 (CDKN2A, **NP_000068**) and overexpression of HER-2/neu (CD340, ERBB2, **NP_001005862**) [21–23]. The *p16* tumor suppressor gene encodes the CDK inhibitor that is involved in the phosphorylation of RB protein, i.e in regulation of the RB-E2F pathway [24–26]. Thus, inactivation of p16 leads to uncontrolled cell growth.

The best prognostic markers for endometrioid carcinoma (type 1 EC) are the high levels of the TP53, ER, and *PTEN* mutations. Other genetic alterations in endometrioid carcinoma include microsatellite instability and specific mutations of *K-RAS* and *β-catenin* genes. β-catenin, a component of the E-cadherin unit of proteins, is essential for cell differentiation, the maintenance of normal tissue architecture, and plays an important role in signal transduction [27–29]. Furthermore, E-cadherin expression occurs in only 62% and 87% of serous and clear cell cancers, respectively. Decreased E-cadherin expression is associated with a loss in cell-cell cohesive forces. E-cadherin-negative tumors are associated with poorer prognosis [30, 31].

In our study, expression of S18-2 and free E2F1 proteins increased significantly in tumor tissue compared to NE an HP samples. This correlates with the fact that S18-2 competes with RB protein for E2F1 binding, thus abolishes hinders in the S-phase entry [10]. As was mentioned in the introduction, overexpression of S18-2 in primary rat cells led to their immortalization and transformation. We have also previously reported that ectopic expression of S18-2 in tumor cell lines, such as breast cancer cell line MCF7 and kidney tumor cells KRC/Y, led to a disturbance in the cell cycle and the formation of multinucleated cells [32]. Interesting question is whether the cytoplasmic and nuclear S18-2 might perform different functions or not. Probably, nuclear S18-2 could be a sign for the worse prognosis, but this needs the further investigation.

The EC HEC-1-A cell line, which overexpresses S18-2 constitutively, showed increased proliferation capacity *in vitro* and *in vivo* (in SCID mice). Moreover, pan-keratin, beta-catenin and E-cadherin signals were diminished in these cells, compared to the parental HEC-1-A line, suggesting that S18-2 promotes epithelial-mesenchymal cell transition (EMT). Increased vimentin signal in HEC-1-A-S18-2 cells, compared with parental line, allows us to draw the same conclusion. Studies on larger number of cell lines are needed to support an idea that the highly expressed S18-2 might be a “surrogate marker” for EMT.

## MATERIALS AND METHODS

### Endometrial tissue samples

Samples were collected from 42 patients with endometrial cancer who underwent surgery at the Department of Women's and Children's Health, Karolinska University Hospital (Stockholm, Sweden). Two separate biopsies were taken from each patient: one from the endometrial tumor tissue and one from the normal endometrium (n=84 samples). In addition, some samples of NE and HP (10 samples of each) were collected by scraping the uterus mucosa. Biopsies were fixed in a neutral buffered 4% formaldehyde solution. After fixation, dehydration, and embedding in paraffin, serial sections were cut at a normal thickness of 5 μm and stained with hematoxylin/eosin for histological diagnosis.

Endometrial adenocarcinomas were graded based on morphological features, according to the criteria of the 2009 International Federation of Gynecology and Obstetrics (FIGO) [11] and placed in six tissue categories: i) highly differentiated adenocarcinoma (HDA, FIGO grade 1); ii) moderately differentiated adenocarcinoma (MDA, FIGO grade 2); iii) minimally differentiated adenocarcinoma (LDA, FIGO grade 3); iv) serous cancers (SC); v) HP; and vi) normal endometrium (NE).

### Antibodies and immunohistochemistry

S18-2 and free E2F1 protein expression was determined by immunohistochemistry of the paraffin-embedded tissue sections. Paraffin was dissolved in xylol and the tissue rehydrated by stepwise washing with EtOH in phosphate-buffered saline (PBS) (99%, 90%, 70%, and 30% EtOH). Tissues were then treated in a 2% solution of H_2_O_2_ in methanol at room temperature for 30 min to reduce background staining. Epitopes were exposed by hot citrate buffer (water bath, 92°C for 15 min). Rabbit polyclonal anti-S18-2 (Proteintech Group, Inc., Chicago, IL, USA) and mouse monoclonal anti-E2F1 (clone 2E10, Sigma-Aldrich, St. Louis, MO, USA) antibodies were diluted (1:200) in blocking buffer (2% bovine serum albumin, 0.2% Tween-20, 10% glycerol, and 0.05% NaN3 in PBS). To stain for free E2F1, the mouse monoclonal antibody raised against the C-terminus of protein was used. It is known that the C-terminal portion of E2F1 (residues 409-426) is involved in binding with RB protein, and therefore in the regulation of the transactivating ability of E2F1 [12, 13]. Protein signals were visualized with the help of the EnVision™ Detection Peroxidase/DAB system (Dako, Glostrup, Denmark). Nuclei were stained with hematoxylin (Dako). Staining was evaluated using a semi-quantitative method, i.e., the number of cells that showed signal was counted. The minimum number of tumor (normal) cells counted was 900.

### Cell culture and immunostaining

The HEC-1-A cell line (ATCC HTB-112) was purchased from ATCC biological resource center (Manassas, VA, USA). This endometrial cell line was isolated from a patient with stage IA EC as described previously [33].

All cell lines were cultured in Iscove's medium, which contained 10% fetal bovine serum and appropriate antibiotics, at 37°C.

The HEC-1-A (1–2·10^4^) cells were transfected with either a plasmid that encodes GFP-fused S18-2 (GFP-S18-2) protein as described previously [10], or with a vector, using Lipofectamine 2000 (Life Technologies, Carlsbad, CA, USA). GFP-plasmid was chosen to achieve the high ectopic expression of S18-2. After 3 weeks in Iscove's medium that contained 0.5 mg/ml G418, transfected cells (clones) were selected. From a 7.5-cm-diameter Petri dish, 6 clones were collected and cultured in six-well plates. Two clones (designated as HEC-1-A-S18-2 in the text) that expressed the highest level of GFP-S18-2 were selected for further experiments. Before immunostaining, cells were grown on cover slips in six-well plates. Cells were fixed in a mixture of cold methanol and acetone (1:1) at −20°C, and prior staining they were rehydrated in PBS. Rabbit anti-S18-2 and anti-beta catenin antibodies (Sigma-Aldrich) and mouse monoclonal anti-E-cadherin, N-cadherin (Cell Signaling Technology, Danvers, MA, USA), anti-keratin and Pan Ab-1 (Thermo Scientific, Waltham, MA, USA), and anti-vimentin (Dako) antibodies were used. Mouse monoclonal anti-cytokeratin 18 was the kind gift of Svitlana Sidorenko (IEPOR of NASU, Kyiv, Ukraine). Rabbit anti-mouse and swine anti-rabbit FITC-conjugated (Dako) sera were used as secondary antibodies. Hoechst 33258 (Sigma-Aldrich) was added at a concentration of 0.4 μg/mL to the secondary antibody for DNA staining. Staining images were captured using a DAS microscope Leitz DM RB with a dual-mode cooled charge-coupled device camera (C4880; Hamamatsu, Japan). Photoshop and Image J software were used to assemble overlay images.

### Cell proliferation assay

Relative proliferation of cells was studied, using the CyQUANT®NF assay (Invitrogen, Carlsbad, CA, USA). Briefly, 500, 1000 and 3000 cells of HEC-1-A and HEC-1-A-S18-2 were grown in triplicates in a 96 well plate overnight in IMDM medium. After medium was removed, an aliquot of fluorescent dye was added and cells were incubated for 60 min at 37°C. After incubation, the fluorescence intensity was measured at λ=530 nm, using microplate reader Omega (BMG Labtech, Ortenberg, Germany). Percentage proliferation was calculated for HEC-1-A-S18-2 in comparison with control cells HEC-1-A, taken as 100%.

### Inoculation of cells in experimental animals, SCID mice

EC HEC-1-A-S18-2 and parental HEC-1-A cells (3×10^6^ cells) were then introduced into the left and right sides of SCID mice; four SCID mice were used for each cell line. All experimental animals were sacrificed at the day 23, when the largest tumor reached 1 cm^3^, as stated in a protocol and approved by the Ethical committee.

### Ethical approvals

Tissue sampling was approved by the Ethics Committee of Karolinska Institutet/Karolinska University Hospital (permit 2006/649), and all women gave written informed consent. Ethical permission to induce tumors in mouse models has also been obtained and is valid for 5 years (Solna court decision number 192/14 from October 9^th^, 2014).

### Statistical analysis

GraphPad Prism software (version 6, GraphPad Software, La Jolla, CA, USA) was used for multiple comparisons of nonparametric criteria. The means of the S18-2 and E2F1 expression (as a percent of positive cells) were analyzed. Further analysis was performed on the combined mean of each set of tumors, according to their grade. A detailed description of the calculations is given in the figure legends.

## CONCLUSIONS

Taken together, our data suggest that a combination of high S18-2 and free E2F1 expression, in parallel with decreased expression of beta-catenin, pan-keratin, and E-cadherin, may be a good set of prognostic markers for EC.

## SUPPLEMENTARY FIGURE


