# Supplementary material for: Mitochondrial ribosomal protein S18-2 is highly expressed in endometrial cancers along with free E2F1
Source: Oncotarget. 2016 Mar 3;7(16):22150–8. doi: 10.18632/oncotarget.7905 (PMC5008351; doi:10.18632/oncotarget.7905)
Supplement: Supplementary file 1 [file oncotarget-07-22150-s001.pdf]

SUPPLEMENTARY FIGURE

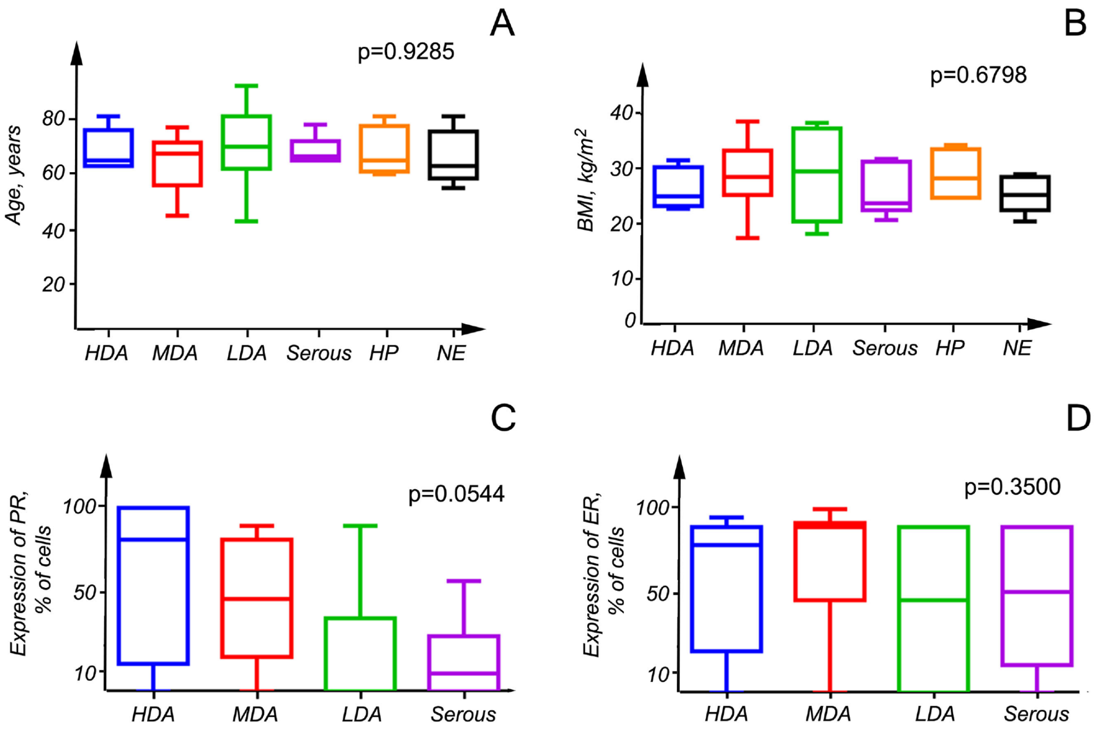

Supplementary Figure S1: Distribution of age and BMI in the study samples and expression levels of progesterone and estrogen receptors by tissue category. Kruskal–Wallis tests were applied to six groups, comprising three of endometrioid adenocarcinomas at various differentiation stages and one serous carcinoma, hyperplasia (HP) and normal endometrium (NE). Age distribution in the groups of patients. No significant differences ($p>0.05$) in age were observed **A.** No significant differences ($p>0.05$) in body mass index were observed in the groups of patients **B.** Kruskal–Wallis tests were applied to four groups, comprising three of endometrioid adenocarcinomas at various differentiation stages and one serous carcinoma. Expression of progesterone receptor (PR) decreased upon tumor progression **C.** No significant differences ($p>0.05$) in high expression of estrogen receptor (ER) was observed in the studied samples **D.**
